# Vertebral artery pseudoaneurysm with recurrent posterior circulation transient ischemic attacks in a post-radiotherapy nasopharyngeal carcinoma survivor: a case report

**DOI:** 10.3389/fonc.2026.1866880

**Published:** 2026-09-04

**Authors:** Haitao Hu, Rui Shang, Yuhu Ma, Meng Ji, Ting Wang, Zexu Wang, Changwei Zhang

**Affiliations:** 1 Department of Neurosurgery, West China Hospital, Sichuan University, Chengdu, Sichuan, China; 2 Clinical Epidemiology and Evidence-based Medicine Center, West China Hospital, Sichuan University, Chengdu, China; 3 NHC Key Laboratory of Clinical Epidemiology and Evidence-based Medicine, West China Hospital, Sichuan University, Chengdu, China; 4 Department of Laboratory Medicine, Zhongnan Hospital of Wuhan University, Wuhan, China

**Keywords:** nasopharyngeal carcinoma, osteoradionecrosis (ORN), radiation-induced osteosarcoma, transient ischemic attack (TIA), vertebral artery pseudoaneurysm

## Abstract

**Background:**

Radiation-induced osteosarcoma (RIOS), osteoradionecrosis (ORN), and post-radiotherapy pseudoaneurysms are recognized late complications of radiotherapy for nasopharyngeal carcinoma (NPC). Although post-radiotherapy pseudoaneurysms usually present with life-threatening hemorrhage, associated ischemic manifestations are rarely described.

**Case presentation:**

A 50-year-old man with a history of NPC treated with radiotherapy developed a left cervical paravertebral RIOS 15 years later, with suspected coexisting cervical spine osteoradionecrosis (C-ORN). He experienced recurrent, brief, and fully reversible posterior circulation transient ischemic attacks (TIAs). Computed tomography angiography (CTA) demonstrated destructive C1–C2 changes, a partially thrombosed left vertebral artery (VA) pseudoaneurysm, and residual distal opacification extending to the V4 segment and left posterior inferior cerebellar artery. Digital subtraction angiography (DSA) performed approximately two weeks later showed no antegrade filling beyond the lesion, while right VA angiography confirmed adequate posterior circulation perfusion. Endovascular parent artery occlusion completely excluded the lesion. No further TIAs or new ischemic complications occurred during the 6-month follow-up.

**Conclusion:**

Post-radiotherapy RIOS, suspected C-ORN, and destructive upper cervical changes may compromise the VA and contribute to pseudoaneurysm formation. Progressive thrombosis of the pseudoaneurysm may be associated with recurrent posterior circulation TIAs. Careful assessment of the vertebral arteries on CTA, supplemented by DSA when indicated, is warranted in patients with otherwise unexplained posterior circulation symptoms.

## Introduction

**Figure 1:**
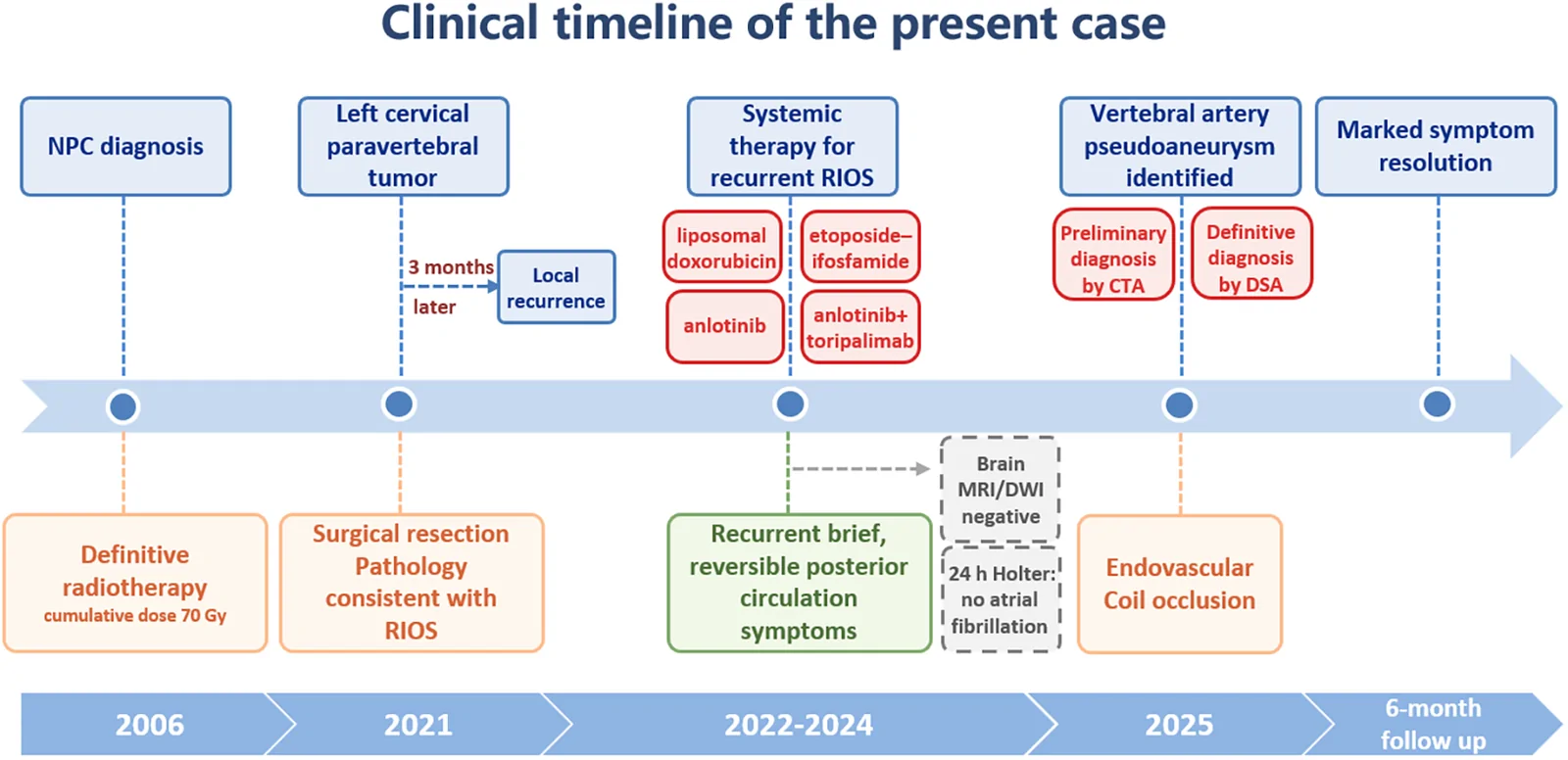
Clinical timeline of the present case. This timeline illustrates the delayed radiation-related oncological and vascular course, from nasopharyngeal carcinoma treatment and radiation-induced osteosarcoma to the development of recurrent posterior circulation TIAs associated with a left vertebral artery pseudoaneurysm. After endovascular coil occlusion, the patient showed No further posterior circulation TIAs occurred during follow-up.

Radiotherapy is central to the management of nasopharyngeal carcinoma (NPC) but may cause late complications, including radiation-induced osteosarcoma (RIOS), osteoradionecrosis (ORN), and vascular pseudoaneurysms (1–3). Radiation-associated pseudoaneurysms usually involve the carotid circulation and present with hemorrhage, whereas vertebral artery involvement and ischemic presentations are rare (2). We report a post-radiotherapy left vertebral artery pseudoaneurysm associated with recurrent posterior circulation transient ischemic attacks (TIAs) in an NPC survivor with RIOS and suspected cervical osteoradionecrosis. Endovascular parent artery occlusion excluded the lesion, and no further TIAs occurred during the 6-month follow-up **Figure 1**.

## Case presentation

**Figure 2:**
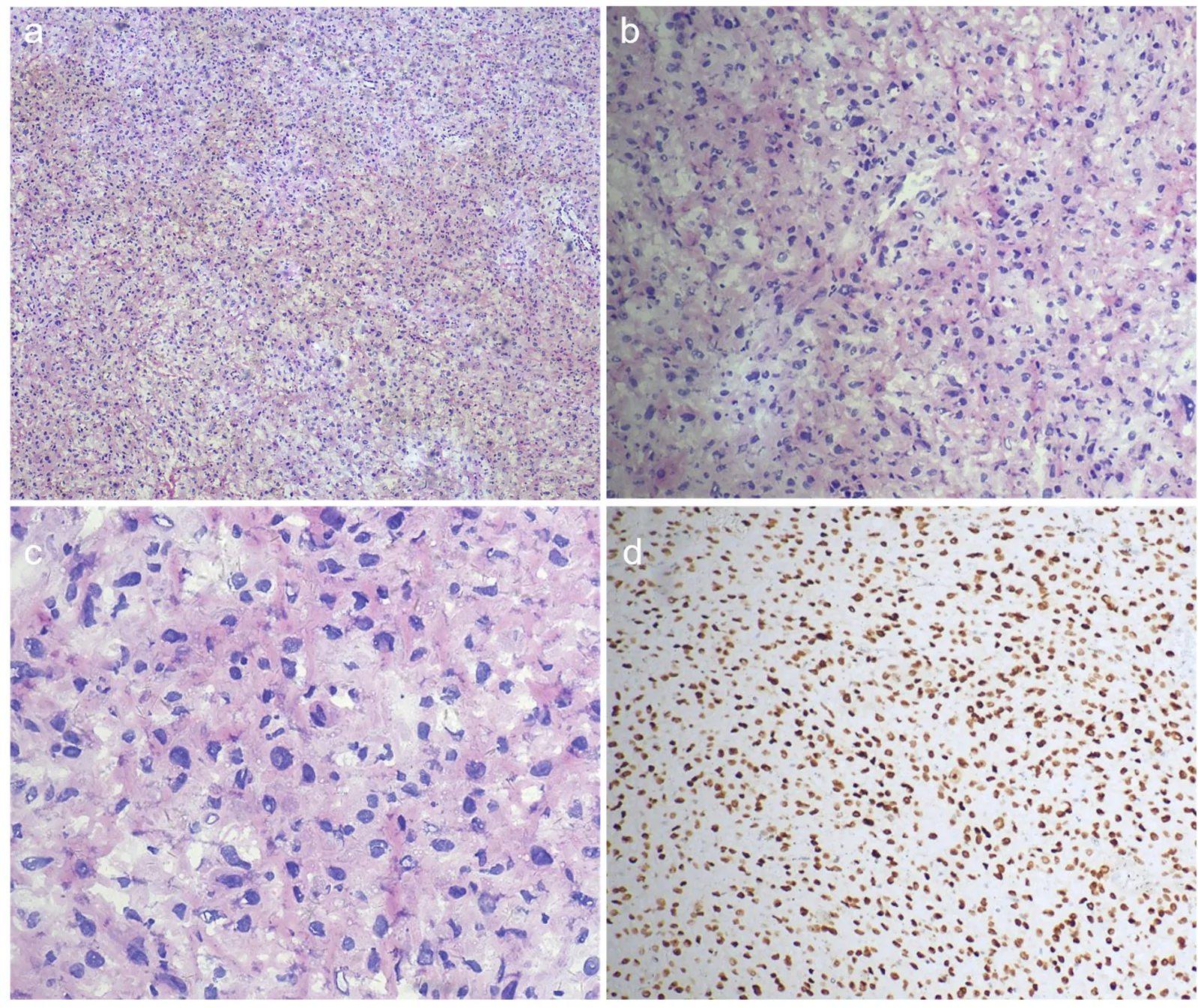
Histopathological and immunohistochemical findings supporting the diagnosis of radiation-induced osteosarcoma. **(a)** Low-power hematoxylin and eosin staining showed a malignant sarcomatous proliferation with heterogeneous cellularity and eosinophilic stromal matrix. **(b, c)** Higher-power views demonstrated pleomorphic short spindle-shaped tumor cells with marked nuclear atypia and hyperchromatic nuclei. **(d)** SATB2 immunohistochemistry showed diffuse nuclear positivity, supporting osteoblastic differentiation. In combination with the long latency after radiotherapy and tumor location within the irradiated field, these findings supported the diagnosis of radiation-induced osteosarcoma. Original magnification: **(a)** [H&E staining, ×40]; **(b)** [H&E staining, ×100]; **(c)** [H&E staining, ×200]; **(d)** [SATB2 immunohistochemistry, ×100].

**Figure 3:**
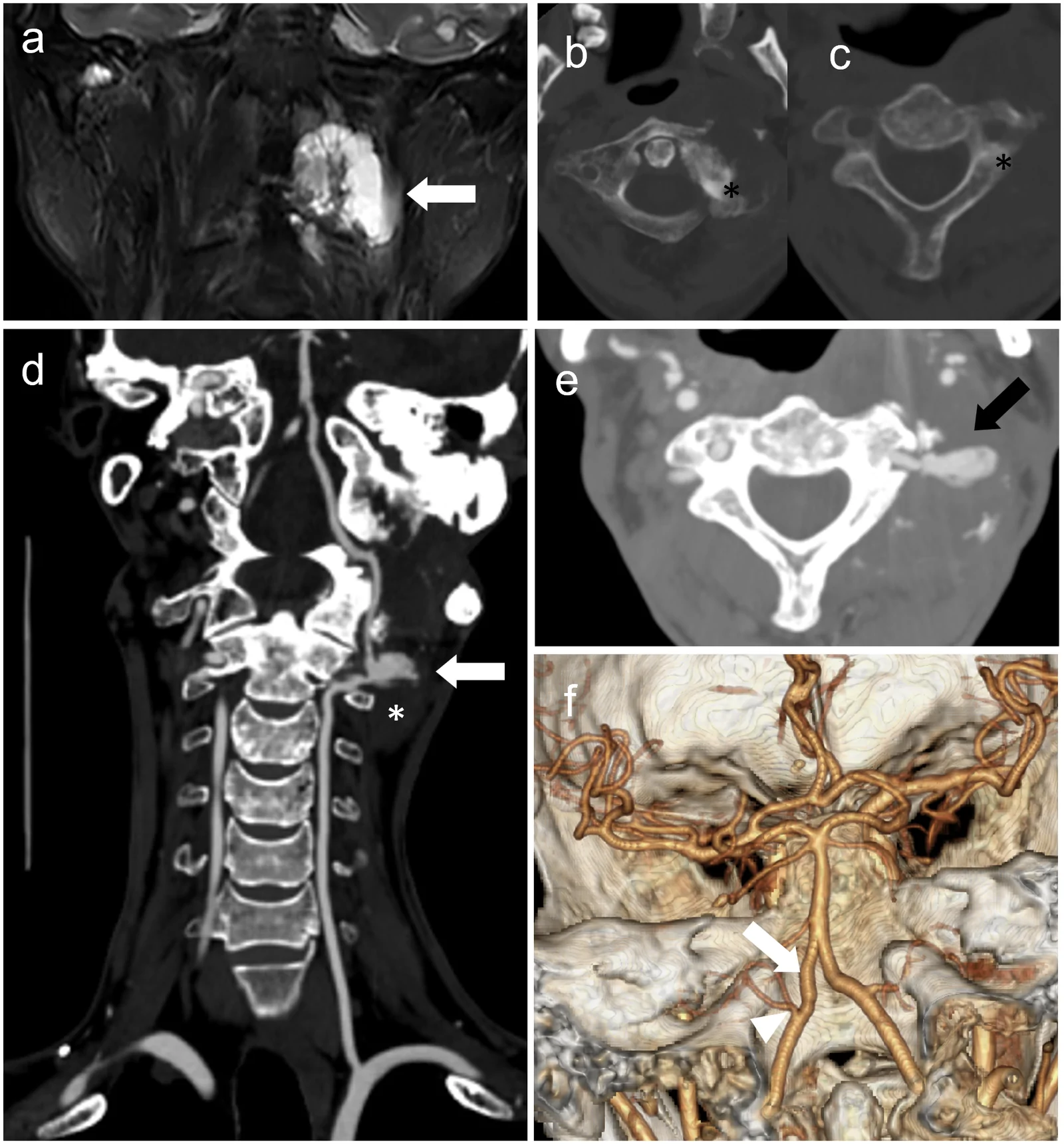
Cervical and vascular imaging after radiotherapy for NPC. **(a)** Coronal MRI showed a heterogeneous left paravertebral mass involving the upper cervical region (white arrow). **(b, c)** Axial CT bone-window images showed mixed osteolytic and sclerotic destruction of the left atlas and axis (asterisks). **(d)** Coronal computed tomography angiography showed the pseudoaneurysm adjacent to the destructive C1–C2 lesion and infiltrative soft-tissue mass (white arrow and asterisk, respectively). **(e)** Axial computed tomography angiography demonstrated an irregular pseudoaneurysm arising from the left vertebral artery (black arrow). **(f)** Posterior three-dimensional computed tomography angiography volume rendering showed opacification of the distal left vertebral artery and left posterior inferior cerebellar artery (arrowhead and arrow, respectively).

A 50-year-old man with a history of NPC, treated with definitive radiotherapy (cumulative dose 70 Gy) in 2006, presented with a left neck mass in 2021. Pathological examination of the left cervical paravertebral tumor revealed an FNCLCC grade 2 malignant spindle-cell sarcoma with osteoblastic differentiation, supported by diffuse SATB2 positivity (**Figure 2**). In the context of the patient’s prior radiotherapy, long latency, and tumor location within the irradiated field, these findings supported the diagnosis of RIOS. Despite surgical resection, local recurrence was detected three months later (**Figure 3a**). He then underwent several lines of systemic therapy, including liposomal doxorubicin, the etoposide–ifosfamide (EI) regimen, anlotinib, and later anlotinib plus toripalimab. He had no history of hypertension, diabetes mellitus, smoking, or alcohol use.

During the treatment course, the patient developed recurrent transient posterior circulation symptoms, including sudden dizziness/vertigo, vomiting, gait instability with veering to one side, and occasional drop attacks. These episodes lasted several minutes and resolved completely without persistent neurological deficits. Dysphagia and choking when drinking liquids were persistent. Cranial magnetic resonance imaging (MRI), including diffusion-weighted imaging, showed no acute infarction, and 24-hour Holter monitoring revealed no atrial fibrillation or other major cardioembolic arrhythmias.

**Figure 4:**
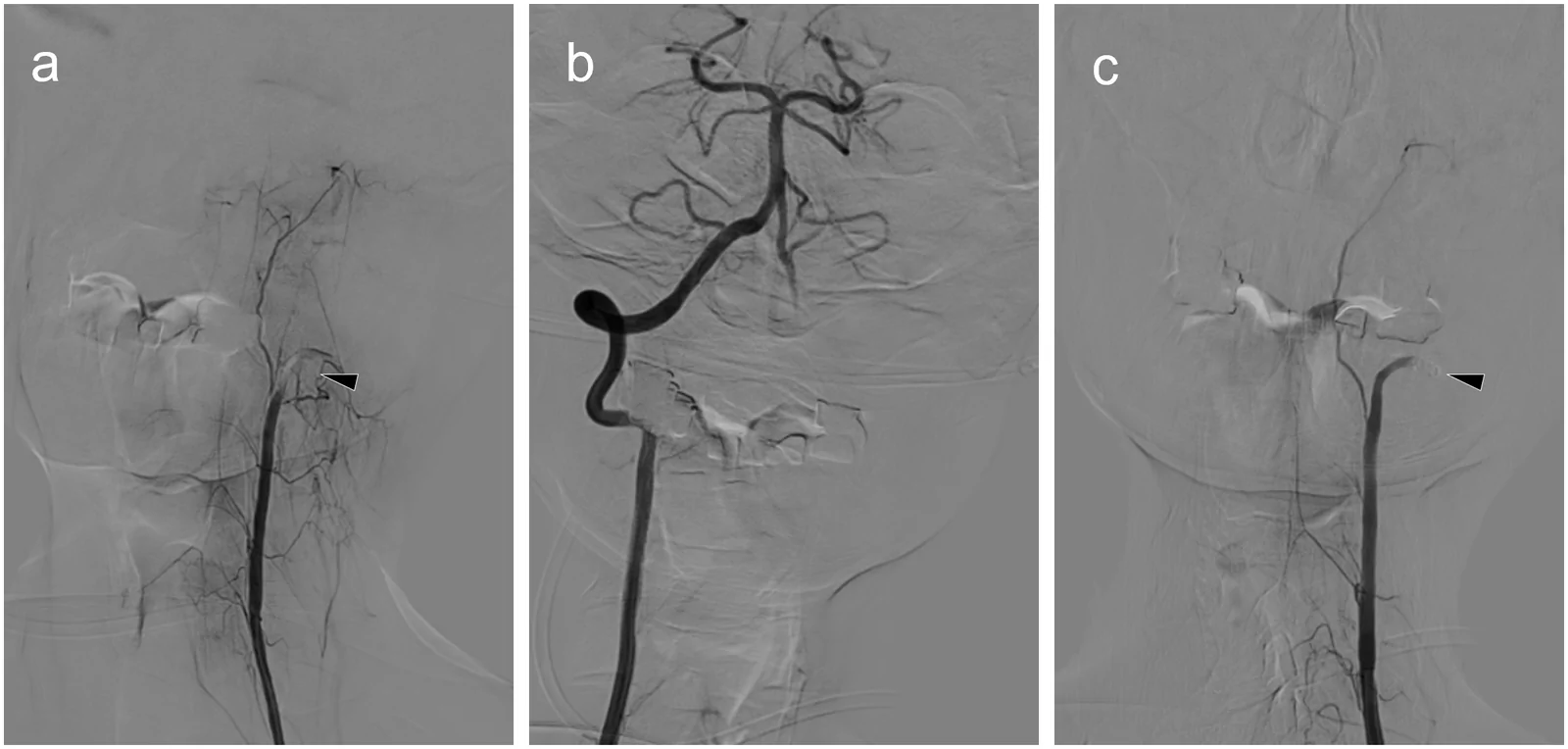
Pre- and post-embolization DSA images. **(a)** Left vertebral artery angiography showing an irregular pseudoaneurysmal stump at the C1–C2 level (arrowhead), with no opacification beyond the lesion. **(b)** Right vertebral artery angiography showing preserved basilar and posterior circulation perfusion, with retrograde opacification of the distal left V4 segment and left posterior inferior cerebellar artery, but not of the pseudoaneurysm. **(c)** Post-embolization angiography confirming complete occlusion of the proximal left vertebral artery (arrowhead), with no residual filling of the pseudoaneurysm.

Computed tomography angiography (CTA) revealed extensive osteolytic destruction of C1–C2, with exposure of the left vertebral artery (VA) and a pseudoaneurysm at the C1–C2 level (**Figures 3b–e**). Contrast opacification extended distal to the pseudoaneurysm through the left V4 segment and into the left posterior inferior cerebellar artery (PICA) (**Figure 3f**). Digital subtraction angiography (DSA) performed approximately two weeks later showed an irregular pseudoaneurysmal stump, with no opacification beyond the lesion on left VA injection. Right VA angiography demonstrated preserved basilar and posterior circulation perfusion, with retrograde opacification of the distal left V4 segment and left PICA, but not of the pseudoaneurysm (**Figures 4a, b**). Before endovascular treatment, the patient had not received antiplatelet or anticoagulant therapy. Laboratory testing showed thrombocytopenia, with a platelet count of 81 × 10^9^/L. Hemoglobin was 149 g/L, coagulation parameters were within normal limits, and D-dimer was 0.48 mg/L FEU. C-reactive protein, white blood cell count, and neutrophil count were also within normal limits. After adequate contralateral vertebrobasilar perfusion had been confirmed, endovascular parent artery occlusion was performed.

Using a 0.035-inch hydrophilic guidewire (Radifocus, Terumo) and a 6-French guiding catheter (100-cm Envoy MPD, Ethicon), a 3 × 3 mm coil (Cook Medical) was deployed to occlude the proximal left VA and exclude the pseudoaneurysm. Post-procedural angiography confirmed complete exclusion of the lesion without residual filling (**Figure 4c**). No further posterior circulation TIAs or new neurological deficits occurred during the 6-month follow-up.

## Discussion

Pseudoaneurysm of the VA represents a rare but devastating sequela of radiotherapy (4). As recognized late-onset complications, pseudoaneurysms, ORN, and RIOS arise from persistent radiation-driven injury to the vasculature, osseous framework, and mesenchymal matrices. Collectively, these pathologies act as the direct and indirect drivers of pseudoaneurysm formation (2).While radiation-associated pseudoaneurysms predominantly affect the carotid system—classified as one of the three subtypes of carotid blowout syndrome (CBS) by Arterio et al.—involvement of the vertebral artery is scarcely reported (2). It is imperative to note that, analogous to carotid lesions, a vertebral artery pseudoaneurysm harbors the potential for catastrophic hemorrhage upon rupture.

To contextualize the present case, we performed a focused PubMed search, for English-language case reports published between 2004 and 2026. Search terms included combinations of “radiotherapy,” “pseudoaneurysm,” “carotid artery,” “vertebral artery,” “nasopharyngeal carcinoma,” “head and neck malignancy,” and “osteoradionecrosis.” Eighteen articles describing 22 post-radiotherapy pseudoaneurysms involving the carotid artery system or vertebral artery were identified (**Supplementary Table 1**). Most lesions involved the internal carotid artery and presented with massive epistaxis or other hemorrhagic events (3). Only two previously reported cases involved the vertebral artery, and both presented with life-threatening hemorrhage rather than posterior circulation ischemic symptoms (4, 5). None of the reviewed cases reported concomitant RIOS. The present case expands the recognized clinical spectrum by describing recurrent posterior circulation TIAs associated with an evolving, partially thrombosed post-radiotherapy vertebral artery pseudoaneurysm.

The etiopathogenesis of RIOS involves a complex interplay between genomic instability and chronic tissue injury, typically manifesting after a median latency of 9 to 12 years (6). In the present case, the diagnosis of osteosarcoma was confirmed pathologically 15 years following a cumulative dose of 70 Gy, strictly satisfying the diagnostic criteria proposed by Cahan and modified by Arlen (6). Concurrently, radiotherapy can induce ORN in any skeletal structure (7). The susceptibility to ORN is intrinsically correlated with skeletal vascularity (7). Unlike the mandible, which is limited by an inherently tenuous blood supply, the cervical spine benefits from a rich vascular network, making osteoradionecrosis of the cervical spine (C-ORN) a comparatively rare entity (8). A comprehensive review by Manson et al (8) revealed that C-ORN typically affects males aged 50.6 to 57.7 years. Key risk factors include NPC treatment and cumulative doses exceeding 60 Gy. Our patient’s clinical and imaging features were consistent with reported characteristics of C-ORN, supporting its consideration as a suspected coexisting pathology. Critically, both RIOS and ORN precipitate extensive osteolytic changes. In this patient, this synergistic destruction compromised the structural scaffold protecting the vertebral artery. Consequently, the vessel was exposed to direct tumor invasion and the indirect vascular toxicity of necrotic bone sequesters, thereby facilitating the formation of the pseudoaneurysm.

The recurrent posterior circulation attacks occurred mainly before CTA, when the residual left VA remained opacified distal to the partially thrombosed pseudoaneurysm, extending to the V4 segment and left PICA. DSA performed approximately two weeks later showed no antegrade filling beyond the lesion, suggesting interval progression to distal occlusion. Distal thromboembolism from the partially thrombosed pseudoaneurysm may therefore have occurred during the earlier partially patent phase (9).

Alternative explanations were considered. Sustained vertebrobasilar hypoperfusion was considered less likely because the right VA provided adequate posterior circulation perfusion and the attacks were unrelated to posture, head movement, exertion, or hypotension. Peripheral vestibular disorders (10), adverse effects of anlotinib-based targeted therapy or toripalimab-based immunotherapy (11), and local tumor- or radiation-related factors could account for some symptoms, particularly dysphagia and choking, but were less consistent with the recurrent, brief, and fully reversible posterior circulation TIAs. Malignancy-associated thrombosis could not be entirely excluded, although no major coagulation abnormality, marked D-dimer elevation, or cardioembolic source was identified.

Given the persistent radiation-related tissue injury, tumor involvement, extensive osteolytic destruction, and suspected coexisting C-ORN, spontaneous stabilization of the pseudoaneurysm was considered unlikely. Because the lesion carried a substantial risk of rupture and might have contributed to the recurrent posterior circulation TIAs, endovascular parent artery occlusion was performed after adequate contralateral vertebrobasilar perfusion had been confirmed. No further posterior circulation TIAs occurred during the 6-month follow-up. Taken together, the temporal imaging changes and post-treatment clinical course support an association between the pseudoaneurysm and the ischemic events, although the exact mechanism remains unproven.

This report has several limitations. Direct evidence of distal microembolism was unavailable. No additional or delayed DSA projections were available to demonstrate a continuous pathway from the pseudoaneurysm to the intracranial circulation. Therefore, a thromboembolic mechanism remains plausible but unproven. The absence of follow-up vascular imaging at 6 months also precluded confirmation of durable occlusion.

## Conclusion

This case highlights a rare delayed vascular complication of radiotherapy for NPC. Pathologically confirmed RIOS, suspected coexisting C-ORN, and extensive upper cervical destruction may collectively compromise the vertebral artery and promote pseudoaneurysm formation. Progressive thrombosis of the pseudoaneurysm may be associated with recurrent posterior circulation TIAs, although the exact thromboembolic pathway remains unproven. In post-radiotherapy patients with destructive upper cervical lesions and otherwise unexplained posterior circulation symptoms, CTA should include careful assessment of the vertebral arteries, with DSA performed when further characterization of lesion patency, collateral circulation, or treatment feasibility is required.

## Data Availability

The original contributions presented in the study are included in the article/**Supplementary Material**. Further inquiries can be directed to the corresponding author.
