# Supplementary material for: Vertebral artery pseudoaneurysm with recurrent posterior circulation transient ischemic attacks in a post-radiotherapy nasopharyngeal carcinoma survivor: a case report
Source: Front Oncol. 2026 Sep 4;16:1866880. doi: 10.3389/fonc.2026.1866880 (PMC13585620; doi:10.3389/fonc.2026.1866880)
Supplement: Supplementary file 1 [file DataSheet1.pdf]

Supplementary Table 1. Summary of published case reports of post-radiotherapy pseudoaneurysms involving the carotid artery system and vertebral artery in patients with head and neck malignancies

Case	Case report	Age/sex	Primary disease/setting	Radiation dose/course	Vessel involved	Presentation	RIOS	ORN	Treatment	Outcome
1	Lo et al., 2025(1)	64/F	NPC (T2N2M0, Stage II); temporal bone/skull-base ORN after radiotherapy	66 Gy	Left superficial temporal artery branch of ECA	Delayed episodic then massive left otorrhagia	NR	+	Selective ECA DSA and coil embolization of superficial temporal artery pseudoaneurysm	Complete pseudoaneurysm occlusion without rebleeding; HBOT chosen for ORN; symptomatic improvement
2	Li et al., 2023, case 1(2)	40/M	NPC (T4N2M0, undifferentiated non-keratinizing carcinoma); recurrence treated with chemotherapy and heavy proton radiation	63 Gy lower neck; 73 Gy upper neck; later heavy proton radiation	Right ICA, rupture-hole/C3 segment	Intermittent nasal bleeding followed by sudden massive nasal hemorrhage (~800 mL)	NR	NR	Stent-assisted coil embolization with balloon-assisted Onyx gel	Discharged without neurological deficits; no further nasal bleeding reported
3	Li et al., 2023, case 2(2)	39/M	NPC (T4N2M0, undifferentiated non-keratinizing carcinoma); pterygoid body and occipital slope invasion	NR	Right ICA C3 initially; recurrent lesion involving C2–C4 segment	Sudden massive nasal bleeding (~800 mL), then recurrent nasal hemorrhage (~500 mL) 3 months later	NR	NR	Initial stent-assisted coil embolization with balloon-assisted Onyx gel; recurrence treated by permanent parent ICA occlusion with coils/Onyx	No further nasal bleeding reported after parent artery occlusion
4	Yuan et al., 2022(3)	36/M	NPC treated with radiotherapy 8 years earlier; no evidence of NPC recurrence on nasoendoscopy	64 Gy	Left ICA, petrous portion/C2 segment	Severe repetitive epistaxis with hemodynamic instability	NR	NR	Heparin-bonded stent graft placement	Hemorrhage controlled without reported complication
5	Park and Jang, 2022(4)	41/M	NPC (T2N2M0) treated with concurrent chemoradiotherapy; recurrence treated with salvage radiotherapy and chemotherapy	67.2 Gy/28 fractions	Right petrous ICA	Massive epistaxis with headache; persistent/intermittent epistaxis after embolization	NR	+	Stent-assisted endovascular coil embolization	Initial angiographic occlusion, but persistent epistaxis; died of hypovolemic shock after massive bleeding during laryngoscopy
6	Lee, 2021(5)	63/F	NPC treated with surgery, radiation, and chemotherapy in 2008	NR	Left ICA, just proximal to mid-petrous segment	Massive epistaxis and syncope	NR	NR	Stent-assisted coiling; repeat/additional coiling for coil compaction and residual leakage	No procedure-related complication; no neurological deficit or epistaxis at 3-month follow-up
7	Ay et al., 2021(6)	61/NR	Recurrent irradiated undifferentiated NPC	NR	ICA at junction of petrous and cavernous segments	Severe recurrent epistaxis despite nasal packing; later nasopharyngeal coil extrusion	NR	+	Coiling and endovascular plug embolization; protruding coil material divided/left in place	Persistent embolization on MRI; no further epistaxis over 9 months; radionecrosis did not heal sufficiently
8	Choby et al., 2019, case 1(7)	64/F	NPC (T4N1M0), disease-free after definitive chemoradiotherapy	NR	ICA	Intermittent minor epistaxis, then massive epistaxis	NR	+	Intervention planned but not performed	Died before emergent intervention
9	Choby et al., 2019, case 2(7)	53/M	NPC after definitive chemoradiotherapy; disease-free with known ORN	NR	Descending palatine branch of left internal maxillary artery (ECA system)	Recurrent epistaxis becoming heavier, requiring ED visits and nasal packing	NR	+	n-butyl cyanoacrylate glue embolization	Complete occlusion; no further epistaxis reported
10	Cheng et al., 2018, case 1(8)	55/M	NPC treated with radiotherapy in 1996; prior right ICA pseudoaneurysm in 2010; later necrotic nasopharyngeal mass/skull-base erosion	NR	Right ICA initially; later left petrous ICA with contralateral ICA already occluded	Massive epistaxis; later profuse oronasal bleeding after biopsy	NR	+	Right ICA coil occlusion initially; later high-flow EC-IC bypass using great saphenous vein graft plus left ICA occlusion	Neurologically intact; asymptomatic 30 months after operation
11	Cheng et al., 2018, case 2(8)	68/M	NPC treated with concurrent chemoradiotherapy 30 years earlier	NR	Left petrous ICA	Massive epistaxis with hemorrhagic shock	NR	NR	High-flow bypass using radial artery graft plus endovascular left ICA occlusion	Neurologically intact; patent bypass at 3 months; neurologically well at 24 months
12	Nazari et al., 2017(9)	52/M	NPC treated with chemotherapy and EBRT; recurrence treated with a second radiation course	NR	Left ICA, just proximal to cervical/petrous segment junction	Recurrent epistaxis, acute worsening epistaxis and syncope; massive epistaxis during admission	NR	NR	Complete left ICA coil embolization	Post-procedure left temporal/parietal infarcts; aphasia at 12-month follow-up
13	Dong et al., 2016(10)	55/M	NPC treated with Gamma Knife radiotherapy 7 months earlier	Central dose 32 Gy; marginal dose 16 Gy	Right ICA at nasopharyngeal level	Severe repetitive epistaxis; acute carotid blowout syndrome	NR	NR	Attempted covered stent failed; emergency right ICA occlusion with coils	Bleeding controlled; some paralysis symptoms; died 40 months later from tumor recurrence without epistaxis
14	Chandran et al., 2016(11)	41/F	Skull-base chordoma excised 20 years earlier with postoperative radiotherapy; delayed radionecrosis	NR	Left vertebral artery, distal V2/proximal V3 region	Recurrent severe life-threatening hemoptysis, including near-fatal bleeding	NR	+	Endovascular occlusion of the left vertebral artery with coils after balloon occlusion/cross-flow assessment	No neurological deficit; no further hemoptysis at 12-month follow-up
15	Hsiao et al., 2014(12)	71/M	NPC treated with radiotherapy 26 years earlier; concomitant ischemic stroke and carotid stenosis	NR	Left high cervical ICA/lower petrous segment	Minor ischemic stroke, recurrent stroke with nasal bleeding, then massive epistaxis with hypovolemic shock	NR	+	Therapeutic left ICA occlusion using microcoils and N-butyl-2-cyanoacrylate glue	Bleeding controlled; consciousness improved but right hemiplegia persisted; tracheostomy required

Case	Case report	Age/sex	Primary disease/setting	Radiation dose/course	Vessel involved	Presentation	RIOS	ORN	Treatment	Outcome
16	Buyukcam et al., 2010(13)	47/M	NPC treated with conventional radiotherapy; second short radiotherapy course for local recurrence	NR	Distal left ICA at nasopharyngeal/skull-base level	Repetitive nasal bleeding progressing to massive epistaxis and hemorrhagic shock	NR	NR	Balloon occlusion of distal and proximal parts of pseudoaneurysm/ICA	Extubated after 6 days; discharged after 28 days; asymptomatic at 7-month outpatient review
17	Cheng et al., 2008(14)	57/M	NPC (T2aN1M0) treated with neoadjuvant chemotherapy and radiotherapy; local recurrence treated with re-irradiation/brachytherapy	70.4 Gy; second course 40 Gy + brachytherapy 18 Gy	Right ICA at nasopharyngeal level	Massive nasal bleeding with repeated bleeding during hospitalization	NR	NR	Attempted embolization with stent grafts, balloons, and bare stents failed; spontaneous self-thrombosis of right ICA	No neurological defects; no nasal bleeding at ≥1.5-year follow-up
18	Li et al., 2007(15)	61/M	NPC (T4N1M0) after concomitant chemoradiotherapy; hypopharyngeal bulging mass mimicking recurrence	63 Gy to head/neck; reduced-field boost 70.2 Gy	Right ECA branch: common trunk of lingual and facial arteries	Sore throat and hypopharyngeal bulging mass; pseudoaneurysm suspected before biopsy	NR	NR	Microcoil embolization of the right lingual/facial artery common trunk	Pseudoaneurysm occluded; biopsies negative for malignancy; mass resolved and sore throat subsided
19	Martinez Del Pero et al., 2006(16)	75/M	Left tonsillar/laryngeal-pharyngeal cancer setting; postoperative radiotherapy 35 years earlier and repeat radiotherapy after recurrence	NR	Left vertebral artery, extracranial course near foramen transversarium/skull base	Catastrophic bleeding through mouth/oropharyngeal ulcer	NR	+	Emergency parent left vertebral artery occlusion with 20 detachable microcoils	PICA infarct and bulbar palsy occurred but resolved; ulcer resolved; stable at 30-month follow-up
20	Chin et al., 2005, case 1(17)	55/M	NPC treated with concomitant chemoradiation 1 year earlier; nasopharyngeal/skull-base necrosis/infection	NR	High cervical left carotid artery/ICA region	Otorrhea/malodorous postnasal drip followed by massive epistaxis	NR	+	Left carotid balloon occlusion	Bleeding stopped, but patient died of meningoencephalitis and sepsis
21	Chin et al., 2005, case 2(17)	59/M	NPC treated 1 year earlier; necrotic/infected nasopharyngeal mucosa	NR	ICA/carotid artery near skull base	Intermittent self-limited epistaxis and halitosis, then fatal massive epistaxis	NR	+	NA; died before intervention	Died at home 2 days after MRI from massive epistaxis
22	Chen et al., 2004(18)	55/M	NPC (T2bN2M0) treated with concomitant radiochemotherapy; admitted for skull-base ORN sequestrectomy	73.8 Gy external beam + intracavitary brachytherapy 8 Gy; 54 Gy lower neck; 73.8 Gy upper neck	Left ICA, distal to carotid bulb before skull base/high cervical region	Bloodstained rhinorrhea followed by sudden massive nasal/oral bleeding and hemoptysis	NR	+	Balloon occlusion/embolization of the left ICA at carotid bulb; endoscopic sequestrectomy	No recurrent cancer on pathology; no bleeding or neurological deficits during 3-month follow-up

Abbreviations: NPC, nasopharyngeal carcinoma; RIOS, radiation-induced osteosarcoma; ORN, osteoradionecrosis; ECA, external carotid artery; ICA, internal carotid artery; VA, vertebral artery; DSA, digital subtraction angiography; EBRT, external beam radiotherapy; EC-IC, extracranial-intracranial; HBOT, hyperbaric oxygen therapy; PICA, posterior inferior cerebellar artery; V2/V3, second/third segment of the vertebral artery; Gy, gray; M, male; F, female; NR, not reported; NA, not available.

Symbols: + indicates reported/present. The classification and wording follow the source articles; NR and NA were used when data were not stated or not available in the original reports.

References for Supplementary Table 1

1. Lo PHW, Kwok HM, Cheng LF, Ma KFJ. Delayed otorrhagia due to radiation-induced external carotid artery pseudoaneurysm complicated by temporal bone osteoradionecrosis in nasopharyngeal carcinoma: A case report and literature review. *Eur Arch Otorhinolaryngol* (2025) doi: 10.1007/s00405-025-09651-y

2. Li C, Lu J, Luo Y, Feng D. Case report: Endovascular intervention of internal carotid artery pseudoaneurysm secondary to nasopharyngeal carcinoma radiotherapy. *Front Surg* (2023) 9:1099416. doi: 10.3389/fsurg.2022.1099416

3. Yuan B, Xin H, Yuan K, Zhang J, Duan F, Wang M-Q. Heparin-bonded stent graft placement for treatment of massive epistaxis from ruptured radiation-induced internal carotid artery pseudoaneurysm: A case report. *Radiology Case Reports* (2022) 17:2129–2132. doi: 10.1016/j.radcr.2022.03.070

4. Park J-S, Jang HG. Endovascular treatment of a ruptured pseudoaneurysm of the internal carotid artery in a patient with nasopharyngeal cancer: A case report. *World Journal of Clinical Cases* (2022) 10:

5. Lee CY. Endovascular treatment for radiation-induced internal carotid artery pseudoaneurysm and usefulness of angiographic and nasal endoscopic confirmation. *Interdisciplinary Neurosurgery* (2021) 24:101031. doi: 10.1016/j.inat.2020.101031

6. Ay N, Todt I, Sudhoff H. Nasopharyngeal coil dislocation of an embolized internal carotid artery pseudoaneurysm. *Case Reports in Otolaryngology* (2021) 2021:1–4. doi: 10.1155/2021/4270441

7. Choby G, Tangbumrungtham N, Dodd R, Patel ZM. Arterial pseudoaneurysm following radiotherapy in patients with a history of nasopharyngeal carcinoma. *OTO Open* (2019) 3:2473974X18823002. doi: 10.1177/2473974X18823002

8. Cheng H-C, Lee C-W, Lee J-E, Tsai J-C, Wang K-C. Emergency high-flow bypass for the management of ruptured postirradiated internal carotid artery pseudoaneurysms in nasopharyngeal carcinoma patients. *Interdisciplinary Neurosurgery* (2018) 14:126–129. doi: 10.1016/j.inat.2018.07.005

9. Nazari P, Tan L, Wewel J, Moftakhar R, Kasliwal M. Massive epistaxis resulting from radiation-induced internal carotid artery pseudoaneurysm. *Neurol India* (2017) 65:380. doi: 10.4103/neuroindia.NI_1259_15

10. Dong F, Li Q, Wu J, Zhang M, Zhang G, Li B, Jin K, Min J, Liang W, Chao M. Carotid blowout syndrome after nasopharyngeal carcinoma radiotherapy: Successful treatment by internal carotid artery occlusion after stent implantation failure. *SpringerPlus* (2016) 5:1553. doi: 10.1186/s40064-016-3209-y

11. Chandran A, Biswas S, Hartley JL, Nahser HC, Et. A. Recurrent life-threatening haemoptysis from a bleeding vertebral artery pseudoaneurysm: A diagnostic dilemma. *Interventional Neuroradiology* (2016) doi: 10.1177/1591019916653248

12. Hsiao C-L, Tsai Y-H, Lin S-K. Massive epistaxis from internal carotid pseudoaneurysm during acute ischemic stroke in a patient with nasopharyngeal carcinoma. (2014) 23:

13. Buyukcam F, Sonmez FT, Aydin K. Successfully treated massive epistaxis in a patient with internal carotid artery pseudoaneurysm. *Journal of Craniofacial Surgery* (2010) doi: 10.1097/scs.0b013e3181e1e834

14. Cheng K-Y, Lee K-W, Chiang F-Y, Ho K-Y, Et. A. Rupture of radiation-induced internal carotid artery pseudoaneurysm in a patient with nasopharyngeal carcinoma—spontaneous occlusion of carotid artery due to long-term embolizing performance. *Head & Neck* (2008) doi: 10.1002/hed.20753

15. Li S-H, Hsu S-W, Wang S-L, Chen H-C, Et. A. Pseudoaneurysm of the external carotid artery branch following radiotherapy for nasopharyngeal carcinoma. *Japanese Journal of Clinical Oncology* (2007) doi: 10.1093/jjco/hym019

16. Pero MMD, Majumdar S, Coley SC, Parker AJ. Near fatal haemorrhage 35 years after radiation for laryngeal cancer: Emergency embolisation of a vertebral artery aneurysm. *Emerg Med J* (2006)

17. Chin S-C, Jen Y-M, Chen C-Y, Som PM. Necrotic nasopharyngeal mucosa: An ominous MR sign of a carotid artery pseudoaneurysm. (2005)

18. Chen H-C, Lin C-J, Jen Y-M, Juan C-J, Et. A. Ruptured internal carotid pseudoaneurysm in a nasopharyngeal carcinoma patient with skull base osteoradionecrosis. *Otolaryngology–Head and Neck Surgery* (2004) doi: 10.1016/j.otohns.2003.08.010
